# Structural analysis and cellular visualization of APP RNA G-quadruplex†
†Electronic supplementary information (ESI) available. See DOI: 10.1039/c9sc02768h


**DOI:** 10.1039/c9sc02768h

**Published:** 2019-10-29

**Authors:** Kaixin Lyu, Shuo-Bin Chen, Chun-Yin Chan, Jia-Heng Tan, Chun Kit Kwok

**Affiliations:** a Department of Chemistry , City University of Hong Kong , Kowloon Tong , Hong Kong SAR , China . Email: ckkwok42@cityu.edu.hk; b School of Pharmaceutical Sciences , Guangdong Provincial Key Laboratory of New Drug Design and Evaluation , Sun Yat-sen University , Guangzhou , 510006 China . Email: tanjiah@mail.sysu.edu.cn

## Abstract

RNA G-quadruplexes (rG4s) are emerging structural motifs that are of pivotal importance in chemistry and biology; however, the current structural information of rG4s is limited, with their folding status and functions in cells remaining elusive. Here, we develop and employ a multi-disciplinary approach to characterize the structure, formation and function of an individual rG4 of interest *in vitro* and in cells. We apply this strategy to a biologically important rG4 in amyloid precursor protein (APP) transcript and reveal distinct structural features of *APP* rG4. Notably, we visualize the formation of *APP* rG4 in cells using an *APP*-specific G-quadruplex-triggered fluorogenic hybridization (GTFH) probe and report that the regulatory role of *APP* rG4 in translation is dependent on rG4 thermostability, providing evidence to the existence and significance of the stable rG4 structure in gene regulation.

## Abstract 2

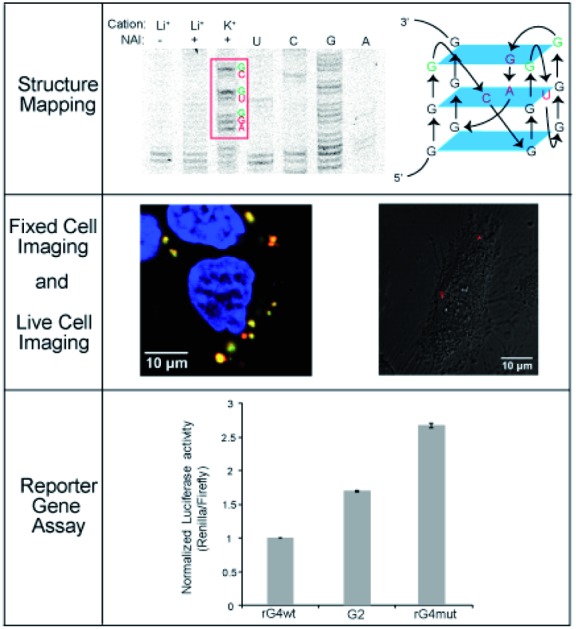
This work introduces a multidisciplinary strategy to characterize the structure, folding status, and function of the RNA G-quadruplex in *APP* 3′UTR.

## Introduction

**Fig. 1:**
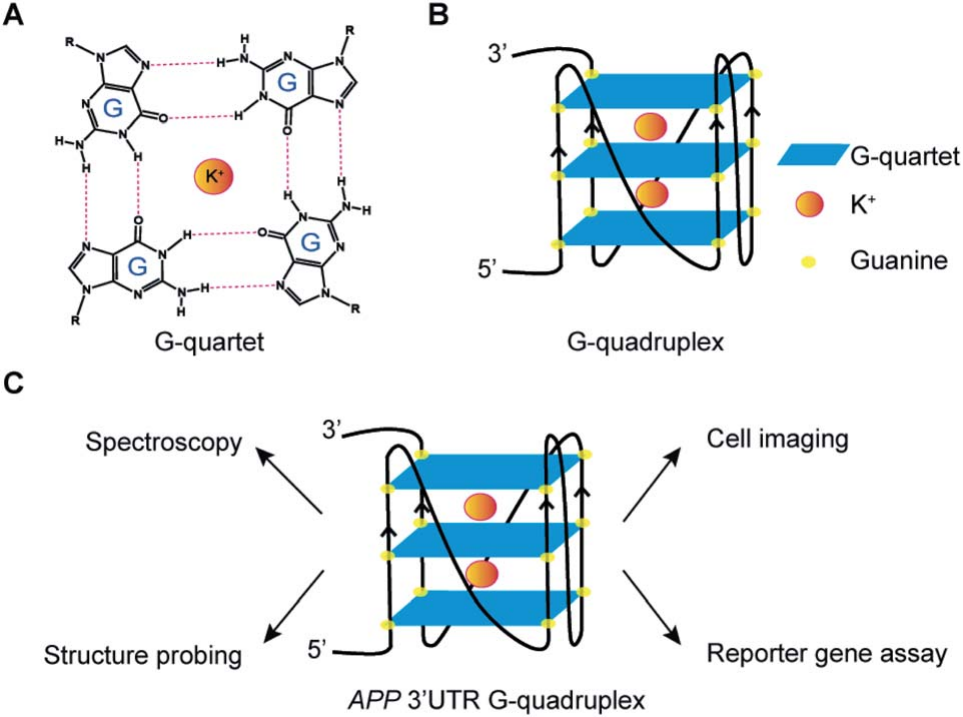
G-quartet, G-quadruplex, and experimental approaches employed. (A) Chemical structure of a G-quartet, potassium ion (K^+^) locates at the centre of the G-quartet for stabilization. (B) G-quartets stack on each other to form the G-quadruplex structure. (C) Multiple assays were employed to investigate the *APP* 3′UTR RNA G-quadruplex formation, structure and function *in vitro* and in cells.

RNA is versatile and folds into diverse structures to govern crucial functions in almost every biological process,^1^,^2^ including transcription, RNA metabolism, and translation. Guanine (G)-rich sequences in RNA can self-assemble into a chemical structure called G-quartet (Fig. 1A) *via* hydrogen bond interactions, which is further stabilized by monovalent cations such as potassium ion (K^+^) and sodium ion (Na^+^), but not lithium ion (Li^+^).^3^–^5^ These G-quartets can stack with each other and are connected by loop nucleotide sequences to form a non-canonical nucleic structure motif referred to as the RNA G-quadruplex (rG4) structure (Fig. 1B).^3^–^5^

The roles of rG4 structures in chemistry and biology are multi-faceted and rapidly emerging, with individual rG4 examples being identified and characterized over the years.^6^–^8^ Transcriptome-wide structure probing studies suggested that rG4s that form *in vitro* are globally unfolded in eukaryotic cells.^9^,^10^ However, latest findings revealed that rG4s are likely dynamic and transient in cells, as they were detected by a live cell imaging G4 probe or pulled down by a biotinylated G4 ligand after cell crosslinking.^11^,^12^ Moreover, rG4 formation in mRNAs, such as at the 5′ and 3′ untranslated region (UTR) of *NRAS*^13^ and *PIM1*,^14^ has been determined to have a suppressive role in translation, whereas rG4 formation in long non-coding RNAs (ncRNA), such as *hTERC*^15^ and *TERRA*,^16^ has been shown to regulate human telomerase RNA maturation and telomere maintenance. Recently, rG4s in short ncRNAs including microRNAs,^17^–^20^ piwi-interacting RNAs,^21^ as well as housekeeping ncRNAs, such as rRNAs^22^ and tRNAs,^23^ have also been reported. In addition, studies have also linked rG4s with diseases such as cancers and neurodegenerative diseases.^8^,^24^,^25^

Amyloid Precursor Protein (APP) is ubiquitously expressed in humans and its proteolytic product amyloid beta (Aβ) can form amyloid plaque, whose accumulation is one of the characteristic pathological features of Alzheimer's Disease (AD).^26^,^27^ We and others have previously identified an rG4 at the 3′UTR in *APP* transcript.^28^,^29^ Nevertheless, the structural characterization of *APP* 3′UTR rG4, especially in the presence of native flanking sequences, is lacking, and the cellular visualization as well as potential mechanism of the inhibitory role of *APP* 3′UTR rG4 in translation remain elusive. In this work, we employ cross-disciplinary methods to characterize the rG4 in *APP* 3′UTR, reporting its structural features, as well as visualizing its formation in cells for the first time (Fig. 1C). Importantly, we further study the effect of *APP* 3′UTR rG4 in translation using dual luciferase reporter gene assays and reveal that the rG4 structure and thermostability can influence translation (Fig. 1C).

## Results and discussion

### Spectroscopic analysis of the *APP* 3′UTR RNA G-quadruplex structure *in vitro*

To characterize the rG4 motif in *APP* 3′UTR, we have initially performed multiple spectroscopic assays. First, circular dichroism (CD) data on the *APP* 3′UTR rG4wt motif (Table S1†), but not on rG4mut motif (Table S1†), showed a positive peak at 262 nm and negative peak at 240 nm, and the CD signal changed upon substituting the K^+^ (G4-stabilizing) with Li^+^ (G4-non-stabilizing) (Fig. S1†). This CD profile and the monovalent-ion dependent feature are indicative of the formation of rG4 with parallel topology.^30^ Second, UV melting data on the *APP* 3′UTR rG4wt motif, but not on the rG4mut motif, exhibited a hyperchromic shift at 295 nm (Fig. S2†), which is a hallmark of rG4 formation.^31^ The melting temperature (*T*_m_) of the *APP* 3′UTR rG4wt motif was determined to be 78 °C under 1 mM K^+^ and >95 °C under 150 mM K^+^ conditions, much higher than the physiological temperature of 37 °C (Fig. S2†). In addition, concentration-dependent UV melting was performed and no change in *T*_m_ was observed from 1 to 10 μM oligonucleotide concentration (Fig. S3†), suggesting that this highly thermostable rG4 is intra-molecularly folded under these testing conditions. Third, intrinsic fluorescence data on the *APP* 3′UTR rG4wt motif, but not on the rG4mut motif, displayed an increase in fluorescence signal upon changing from Li^+^ to K^+^ (Fig. S4†), which is in agreement with the intrinsic fluorescence properties of rG4 reported by our group recently.^32^ These results indicate that the rG4 motif in *APP* 3′UTR exhibits parallel topology, is thermostable, intramolecularly folded, and can exhibit intrinsic fluorescence at physiologically relevant K^+^ concentration and temperature.

**Fig. 2:**
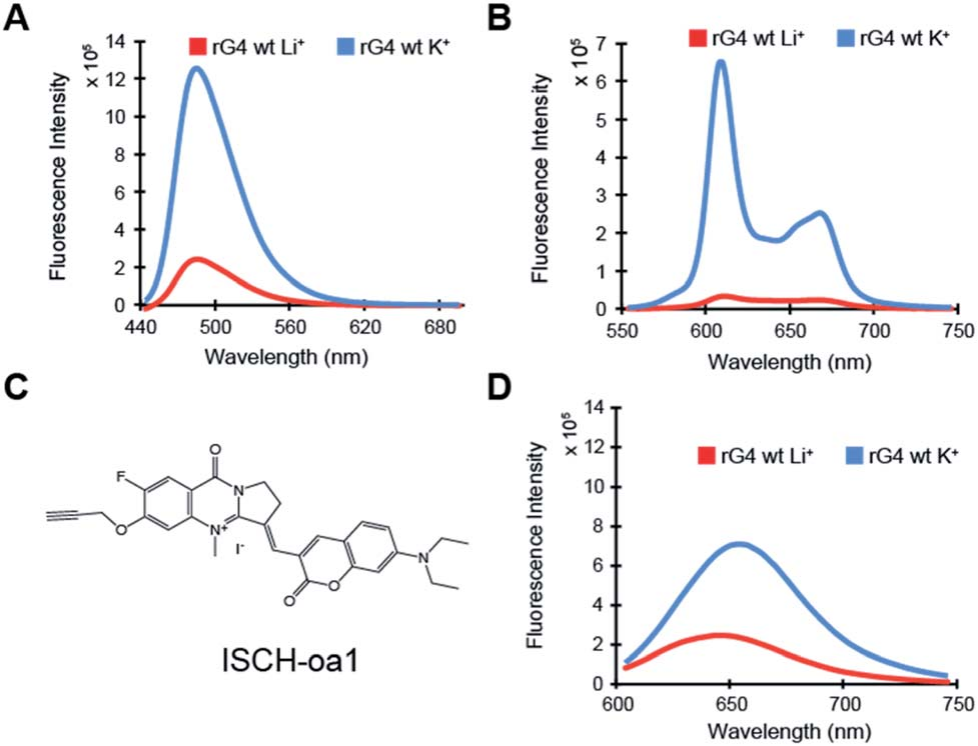
Fluorescence turn-on assays reveal formation of rG4 in the *APP* 3′UTR rG4wt region. (A) ThT ligand-enhanced fluorescence on the *APP* 3′UTR rG4wt region in 150 mM LiCl and KCl, respectively. Spectra under KCl condition showed a positive 5-fold difference in fluorescence (at 494 nm), compared to its LiCl counterpart. (B) NMM ligand-enhanced fluorescence on the *APP* 3′UTR rG4wt region in 150 mM LiCl and KCl, respectively. Spectra under KCl condition showed a positive 19-fold difference in fluorescence (at 614 nm), compared to its LiCl counterpart. (C) Chemical structure of ISCH-oa1. (D) ISCH-oa1 ligand-enhanced fluorescence on the *APP* 3′UTR rG4wt region in 150 mM LiCl and KCl respectively. Spectra under KCl condition showed a positive 3-fold difference in fluorescence (*ca.* 650 nm), compared to its LiCl counterpart.

Next, we aim to evaluate the formation of *APP* 3′UTR rG4 in the presence of flanking sequences, as there are so far only limited rG4 studies that consider sequences beyond the rG4 motif itself.^5^,^33^ To do this, we have introduced some native *APP* 3′UTR sequence to our constructs and designed both rG4wt and rG4mut versions (Table S1†). We first tested these longer constructs, namely the *APP* 3′UTR rG4wt region and *APP* 3′UTR rG4mut region (Table S1†), by employing fluorescence turn-on assays with G-quadruplex-specific ligands such as ThT and NMM. Our results showed that for both ThT and NMM, strong fluorescence enhancement was observed for the *APP* 3′UTR rG4wt region only when changing from Li^+^ to K^+^ (Fig. 2A and B), whereas similar fluorescence intensity was observed for the *APP* 3′UTR rG4mut region under both Li^+^ and K^+^ conditions (Fig. S5A and B†), suggesting the formation of the rG4 structure in the presence of flanking sequences, which allows for G4 ligand recognition and thus increases ThT or NMM fluorescence intensity. These spectroscopic features are consistent with our recently reported ThT and NMM data on rG4-containing short microRNAs,^19^ and our data presented here further extended the application of these assays to the study of rG4-containing long RNAs. Notably, we have also utilized our recently reported G4 fluorescent probe ISCH-oa1 (Fig. 2C),^34^ and demonstrated that it can also detect rG4 formation in the *APP* 3′UTR rG4wt region (Fig. 2D), but not rG4mut region (Fig. S5C†), highlighting that this rG4 detection strategy can be generally applied to different fluorescent turn-on G4 ligands.^35^

### Structural analysis of *APP* 3′UTR RNA G-quadruplex structure *in vitro* and in cell lysate

**Fig. 3:**
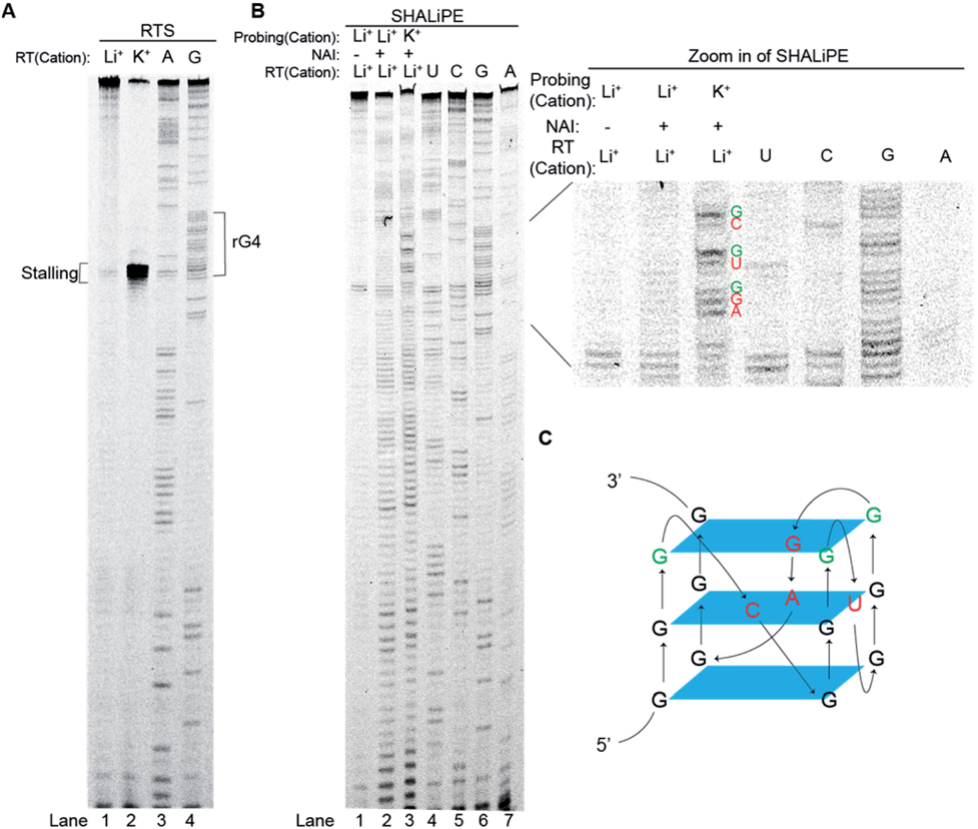
RTS and SHALiPE assays report distinct structural features of rG4 in the *APP* 3′UTR rG4wt region. (A) RTS assay on *APP* rG4wt. The labeled G-rich sequence is the four G-tracts from the *APP* 3′UTR rG4wt region. In lane 1 and 2, 150 mM Li^+^ and 150 mM K^+^, respectively, were used for reverse transcription. Lanes 3 and 4 are ladders generated by dideoxynucleotide sequencing. The stalling site was at the 3′ end of the rG4. (B) SHALiPE assay on the *APP* 3′UTR rG4wt region. The *APP* 3′UTR rG4wt region was probed with DMSO (control) or NAI under Li^+^ and K^+^ conditions (lanes 1–3). The rG4 at the *APP* 3′UTR rG4wt region displayed distinct NAI profiles between Li^+^ and K^+^ conditions (lanes 2–3). On the right is the zoom in of the rG4 motif from the SHALiPE gel. From the band intensity, the nucleotides of the loops of rG4 (red), and the G nucleotides at the 3′ of each G-tract were highly modified (green). (C) Proposed *APP* 3′UTR rG4 structure, with loop nucleotides labeled red and 3′ G of G-tracts labeled green.

To investigate further on the physical location of the rG4 structural motif in the *APP* 3′UTR rG4wt region, we have carried out a reverse transcriptase stalling (RTS) assay, originally developed by us,^36^ under Li^+^ and K^+^ conditions (Fig. 3A). Our results showed that only low RTS was observed under Li^+^ condition, while a strong stalling was presented 3′ proximal to the rG4 motif in the *APP* 3′UTR rG4wt region under K^+^ condition (Fig. 3A), suggesting that the reverse transcriptase was stalled by the *APP* 3′UTR rG4 structure. This stalling is mediated by the presence of thermostable rG4, and in this case *T*_m_ is 78 °C (1 mM K^+^) and >95 °C (150 mM K^+^), as evidenced by our UV melting data on the *APP* 3′UTR rG4wt motif (Fig. S2†). We also performed an identical experiment on the *APP* 3′UTR rG4mut region and found that no stalling was observed for both Li^+^ and K^+^ conditions, verifying that the RTS is caused by rG4 (Fig. S6†). These findings revealed the exact location of the rG4 in the *APP* 3′UTR rG4wt region and provided solid support to the spectroscopic data presented above.

To examine the rG4 structural feature at single nucleotide resolution, we have employed selective hydroxyl acylation analyzed by lithium-ion mediated primer extension (SHALiPE),^18^ which utilizes 2-methylnicotinic acid imidazolide (NAI) to modify flexible RNA nucleotides, followed by reverse transcription using our homemade lithium-based buffer (to avoid RTS from occurring) to detect the NAI-modified nucleotides (Fig. 3B).^18^ Our data indicated that the NAI modification pattern is different between Li^+^ and K^+^ conditions for the *APP* 3′UTR rG4wt region (Fig. 3B), but not for the rG4mut region (Fig. S6†). A closer examination revealed that the difference is mainly at the rG4 region (Fig. 3B, zoom in), suggesting that the formation of rG4 causes local RNA structural rearrangement only. Specifically, the nucleotides of the loops of rG4 (Fig. 3B, red), and the G nucleotides at the 3′ of each G-tract were highly modified (Fig. 3B, green). This specific NAI modification pattern resembled our previous findings on several RNAs such as *pre-miRNA149*, *TERRA* and *hTERC*,^18^ highlighting that this NAI modification profile may be a distinctive structural feature for rG4 identification in the RNA structure probing assay. Using the SHALiPE data, we have identified the loop nucleotides (L1: C, L2: U, L3: GA) and Gs (3 G-quartet planes) involved in the formation of *APP* 3′UTR rG4 (Fig. 3C), providing *APP* 3′UTR rG4 structural information at single nucleotide resolution for the first time. To make our structural findings more biologically relevant, we have also performed SHALiPE assay in cell lysate (Fig. S7†), and found that the NAI modification pattern is largely similar to the K^+^ condition rather than the Li^+^ condition *in vitro* (compare Fig. 3B and S7†), supporting the formation of *APP* 3′UTR rG4 conformation under physiologically relevant conditions.

Collectively, our biophysical characterization and structural analysis have offered substantial evidence for the formation of rG4 in a construct that contains native *APP* 3′UTR sequences *in vitro* and in cell lysate. Notably, our findings on the *APP* 3′UTR rG4 structure are new and the distinct rG4 spectroscopic and structural profiles revealed can likely be used as general structural signatures to identify and characterize rG4s in different contexts.

### Visualization of the *APP* 3′UTR RNA G-quadruplex structure in human cells

**Fig. 4:**
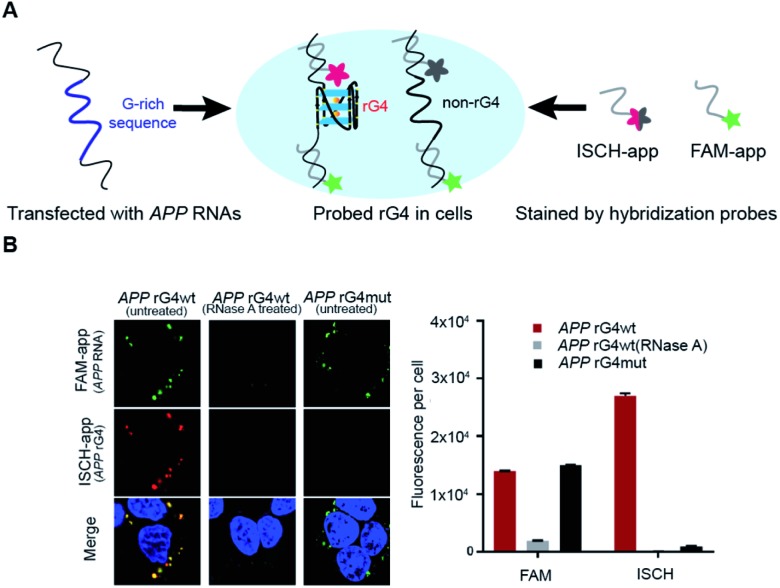
Cell imaging shows *APP* 3′UTR rG4 formation in HEK293T cells. (A) Scheme of the design and utilization of hybridized probes for rG4 visualization. *APP* 3′UTR RNA was first transfected. FAM-app probe in green and ISCH-app probe in red were then used for cell staining, simultaneously. (B) Cell imaging result of transfected *APP* rG4wt and mutant region. Left shows the imaging result under a confocal microscope for both FAM-app probe and ISCH-app probe. RNaseA was added for control. On the right is the quantification of ISCH-app spots and FAM-app spots in cells. The data were acquired from 10 000 cells per sample and three independent experiments were performed.

To explore the folding status of *APP* 3′UTR rG4 in cells, we next attempted to visualize its formation in HEK293T cells (Fig. 4A). To do this, the *APP* 3′UTR rG4wt and rG4mut regions were transfected independently into the cells. The cells were then fixed using paraformaldehyde, followed by staining of two probes, namely ISCH-app and FAM-app (Table S1 and Fig. S8†). ISCH-app is a G-quadruplex-triggered fluorogenic hybridization (GTFH) probe that is made up of two parts (Fig. S9 and 10†): the first part is a fluorescent light-up moiety specific to G-quadruplex structures, and the second is an antisense DNA molecule that can hybridize to the *APP* 3′UTR sequence that is adjacent to the rG4 motif. The FAM labeled antisense oligonucleotide probe was used to track the transfected RNA as well as to normalize the difference in transfection efficiency. Interestingly, we have identified three key observations from the cellular imaging experiments.

First, we found that the *APP* 3′UTR rG4wt region showed green fluorescence, which suggested the successful transfection of corresponding RNA and hybridization of FAM-app probes in cells (Fig. 4B). Notably, red fluorescence was also observed in this construct (Fig. 4B), indicating the formation of *APP* 3′UTR rG4 in cells, as ISCH-app is both sequence and rG4 specific. Merging of the FAM and ISCH fluorescence imaging data revealed a good overlap in signals (Fig. 4B), illustrating that the rG4 detected was from the transfected *APP* 3′UTR rG4wt region RNA.

Second, RNase control was performed on the *APP* 3′UTR rG4wt region, and both the green and red fluorescence were greatly reduced (Fig. 4B), indicating the digestion of transfected RNA and thus less hybridization of the FAM-app and ISCH-app probes in cells. The imaging experiments were also performed on the *APP* 3′UTR rG4mut region, and we found that green fluorescence was detected (Fig. 4B), showing the successful transfection of corresponding RNA and hybridization of FAM-APP probes in cells, however, very low red fluorescence was detected (Fig. 4B), suggesting no rG4 formation in the *APP* 3′UTR rG4mut region. This in-cell result is in line with the *in vitro* fluorescence turn-on and structure probing data (Fig. 2 and 3), supporting rG4 formation in the *APP* 3′UTR rG4wt region.

Last, we have also performed these experiments in HeLa cells (Fig. S11†) and found a similar trend to HEK293T cells (Fig. 4B). In particular, we have quantified the fluorescence per cell, and found that for HEK293T cells, while the FAM signal is about the same between the *APP* 3′UTR rG4wt and rG4mut regions, the ISCH signal is about 28-fold higher in the *APP* 3′UTR rG4wt region (Fig. 4B). Similarly, we observed a 50-fold higher signal for the *APP* 3′UTR rG4wt region *versus* rG4mut region in HeLa cells (Fig. S11†), suggesting that the results observed are independent of cell lines. Moreover, we also performed the imaging experiments in live HeLa cells and observed fluorescence turn-on only in the *APP* 3′UTR rG4wt region but not in the rG4mut region (Fig. S12†), providing strong evidence for rG4 formation in live cells.

### Gene regulatory role of the *APP* 3′UTR RNA G-quadruplex structure in human cells

**Fig. 5:**
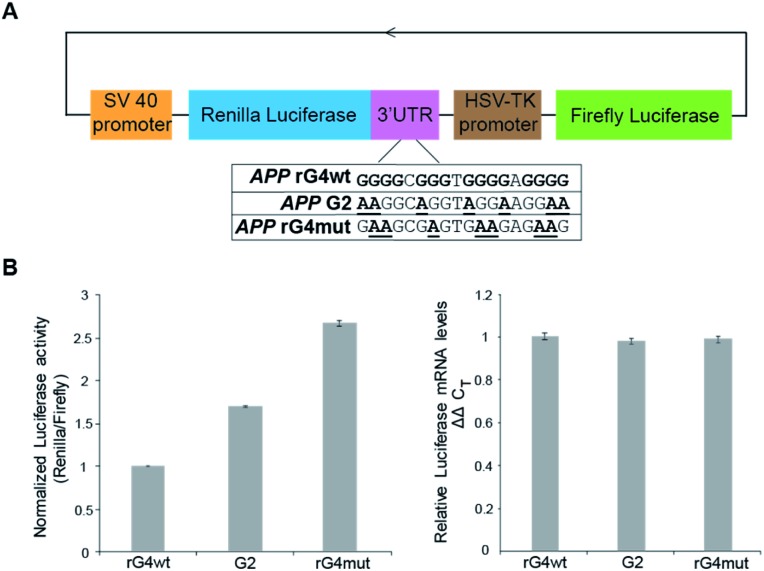
Reporter gene assays uncover the gene regulatory role of the *APP* 3′UTR rG4 structure and stability in HEK293T cells. (A) Scheme of the design of reporter gene plasmids, and sequences of the rG4 wildtype (wt), rG4 partial mutant (G2), and rG4 mutant (mut). (B) Reporter gene assay result on *APP* rG4wt, G2 and rG4mut plasmid. Average normalized luciferase activity was acquired two days after transfection. On the right is the qRT-PCR result on *APP* rG4wt, G2 and rG4mut plasmid, respectively. Values were obtained from 3 biological replicates and error bars display standard error of mean.

To study the role of the *APP* 3′UTR rG4 structure in gene regulation, we have first generated a reporter plasmid that contains the corresponding *APP* wildtype rG4 site in the 3′UTR of the Renilla luciferase gene sequence, as well as a separate plasmid that contains the *APP* mutated rG4 site (Fig. 5A). Dual luciferase reporter gene assays were performed independently on each plasmid (*APP* rG4wt and *APP* rG4mut) in HEK293T cells. Our results showed that compared to the rG4 mutant construct, the rG4 wildtype construct has a 2.7-fold lower normalized luciferase activity (Fig. 5B), suggesting that *APP* 3′UTR rG4 negatively regulates the gene expression. The mRNA level was also measured by the qRT-PCR assay, and no significant changes were observed between the two constructs (Fig. 5B), highlighting that *APP* 3′UTR rG4 controls at the translational level, which is consistent with our prior findings on HEK293T cell lines,^28^ regardless of the fact that the rG4 mutant used in that study was different. To strengthen and extend our findings here, we have performed the same assays in HeLa cell lines to test if the same rG4 effect can be observed. Our results suggested that 6-fold lower normalized luciferase activity was found in the *APP* 3′UTR rG4wt construct when compared with the rG4mut construct (Fig. S13†), and qPCR results showed no significant difference in mRNA level (Fig. S13†), indicating that the suppressive role of *APP* 3′UTR rG4 in translation is conserved in both HEK293T and HeLa cells.

To test whether the thermostability of rG4 can contribute to the inhibition of translation observed above, we have designed an additional rG4 partial mutant (*APP* G2) that changed *APP* 3′UTR rG4 from three G-quartet planes in the wildtype into rG4 with only two G-quartet planes (Fig. 5A), and performed dual luciferase reporter gene assays. Our results in HEK293T cells showed that the luciferase activity of the *APP* G2 construct is about 1.7-fold higher than that of the *APP* rG4wt construct, but 1.6-fold lower than that of the *APP* rG4mut construct (Fig. 5B), supporting our hypothesis. The same phenomenon was also observed in HeLa cells (Fig. S13†). To validate our results, we have also shown the formation of this rG4 motif in *APP* G2 using CD and fluorescence assay, and determined the *T*_m_ of the rG4 motif in *APP* G2 to be 46 °C using UV melting at 150 mM K^+^ (Fig. S14†), which is lower than that of the *APP* rG4wt motif (Fig. S2†). The effect of rG4 thermostability observed in the reporter gene assay is in line with a previous study on an rG4 structure at the 5′UTR of a proto-oncogene *NRAS*,^37^ suggesting that this phenomenon may be general and our luciferase data can correlate with the rG4 structure and its thermostability.

Overall, our cellular imaging and reporter gene analysis have provided solid support for the formation and role of *APP* 3′UTR rG4 in both HEK293T and HeLa cells. Importantly, we uncovered the relationship between rG4 thermostability and translation in *APP* 3′UTR, which sheds light on the better understanding and controlling of the *APP* 3′UTR rG4-mediated translational regulation network in the future.

Recent transcriptome-wide studies have identified many potential rG4s at the 3′UTR,^29^ of which several individual candidates were previously characterized to have a regulatory role in translation,^6^,^8^,^38^ such as *PIM1*.^14^ Our results in *APP* 3′UTR rG4 suggested its suppressive role in translation, and we further reinforced the reporter gene data by characterizing the *APP* rG4 structure *in vitro* and visualizing the *APP* rG4 formation in both HEK293T and HeLa cells for the first time. It's worth noting that rG4 might play a stronger or weaker role in the context of the native transcript. As studies have shown that the level of APP protein expression resulted in negative changes in neuronal structure and function,^26^,^39^ it will be of great interest to identify the molecular mechanisms affecting APP expression, which could potentially contribute to the controlling and prevention of Alzheimer's disease. Although the regulatory elements and interacting partners that interplay with the *APP* 3′UTR rG4 structure are still largely unknown and under investigation, we have provided some new and interesting data here that suggest that translation can be controlled by the rG4 structure formation and thermostability. Our findings here offer an important first step to delineate the biochemical mechanism that governs *APP* 3′UTR rG4-mediated translational repression.

## Conclusions

In sum, our structural analyses using spectroscopic, RTS and SHALiPE assays have revealed the structural features of *APP* 3′UTR rG4 at nucleotide resolution for the first time. Importantly, our cellular assays using GTFH probes and reporter gene assays have uncovered the formation and role of *APP* 3′UTR rG4 in cells. The strategies and findings presented here should enable the study of rG4s in any RNA classes and explore the rG4 structure–function relationship in different organisms.

## Experimental

### Preparation of DNA and RNA oligonucleotides for spectroscopy and luciferase assays

DNA and RNA oligonucleotides investigated in this study were purchased commercially (Integrated DNA Technologies, USA and BGI, China). Vectors used in the dual luciferase reporter gene assay were synthesized by iGENE (China).

### Preparation of *in vitro* transcribed (IVT) RNAs for spectroscopy, structure probing and cell imaging experiments

IVT RNAs were prepared using DNA hemi-duplex (Integrated DNA Technologies, USA) and HiScribe T7 High Yield RNA Synthesis Kit (New England Biolabs, USA). RNA was purified using precast 15% denaturing polyacrylamide gel and the gel band with the desired RNA product was cut under UV. The gel was then crushed and soaked in 1× 10 mM Tris–HCl pH 7.5, 1 mM EDTA, and 800 mM LiCl (1× TEL800) in a thermoshaker at 4 °C and 1300 rpm overnight, and the RNA was gel extracted, purified and concentrated using RNA Clean & Concentrator-5 (Zymo Research, USA). RNA was suspended in 20 μL water and kept at –20 °C.

### Circular dichroism (CD) spectroscopy

CD spectroscopy was conducted using a Jasco J-1500 CD spectrophotometer, and a 1 cm path length quartz cuvette (Hellma Analytics) was employed in a volume of 2 mL. Samples with 5 μM RNA (final concentration) were prepared in 10 mM LiCac (pH 7.0) and 150 mM KCl/LiCl. Each of the RNA samples was then thoroughly mixed and denatured by heating at 95 °C for 5 minutes and cooled to room temperature for 15 minutes for renaturation. RNA samples were excited and scanned from 220 to 310 nm at 25 °C and spectra were acquired every 1 nm. All spectra reported were average of 2 scans with a response time of 2 s nm^–1^. They were then normalized to molar residue ellipticity and smoothed over 5 nm.^32^ All data were analyzed with Spectra Manager Suite (Jasco Software).

### Thermal melting monitored by UV spectroscopy

Samples were prepared to a concentration of 10 mM LiCac buffer, 150 mM KCl (unless otherwise stated) and 5 μM RNA, with a total volume of 2 mL. For the concentration dependent experiments, the samples were prepared with RNA concentrations ranging from 1 to 10 μM. Each of the samples was mixed thoroughly and heated at 95 °C for 5 minutes to denature the RNA. It was then cooled for 15 minutes at room temperature for renaturation. All UV melting experiments were performed on an Agilent Cary 100 UV-vis Spectrophotometer, using a 1 cm path length quartz cuvette. Before the experiment started, the sample block was first flushed with dry N_2_ gas and cooled down to 5 °C for 5 minutes. After the sample solutions were loaded to the cuvettes, they were sealed with 3 layers of Teflon tape to prevent vaporization at high temperature. The samples were scanned from 5 to 95 °C with a temperature increment rate of 0.5 °C min^–1^. The temperature was held at 95 °C for 5 minutes before a reversed scan was performed, scanning from 95 to 5 °C with a rate of 0.5 °C min^–1^. The unfolding and folding transitions in both scans were monitored at 295 nm. From the raw data obtained we subtracted data of the blank solutions, which contained the same concentrations of LiCac buffer (pH 7.0) and corresponding salt solution only. It was then smoothed over 10 data points and its first derivative was plotted in Microsoft Excel.^32^ The final melting temperature was obtained by averaging the melting temperatures in the forward and reversed scans.

### Fluorescence spectroscopy

Fluorescence spectroscopy was performed using a HORIBA FluoroMax-4, and a 1 cm path length quartz cuvette (Hellma Analytics) was used with a volume of 2 mL. Samples with 5 μM DNA/RNA were prepared in 10 mM LiCac (pH 7.0) and 150 mM KCl/LiCl. The samples were then denatured at 95 °C for 5 minutes and cooled to room temperature for 15 minutes for renaturation. For the measurement of the intrinsic fluorescence of G-quadruplexes, the samples were excited at 260 nm and the emission spectra were acquired from 300 to 500 nm. Spectra were acquired every 2 nm at 25 °C. The bandwidth of the entrance and exit slits was 5 nm. All data were smoothed over 5 nm. Results here are from three independent experiments and analyzed using Microsoft Excel.

### Preparation of chemical ligands

*N*-Methyl-mesoporphyrin IX (NMM) and Thioflavin T (ThT) were purchased from Frontier Scientific and Solarbio Life Sciences, respectively. ISCH-oa1 was produced based on a scheme published before.^34^ The ligands were all dissolved in DMSO before use.

### Ligand-enhanced fluorescence

Sample solutions containing 0.5 μM RNA were prepared in 150 mM LiCl/KCl, 10 mM LiCac buffer (pH 7.0) and 0.5 μM ligand (NMM/ThT/ISCH-oa1). Fluorescence spectroscopy was performed using a HORIBA FluoroMax-4, and a 1 cm path length quartz cuvette (Wuxi Jinghe Optical Instrument Co.) was used with a sample volume of 100 μL. Before the measurement, the samples (ligand not added) were denatured at 95 °C for 3 minutes and allowed to cool down at room temperature for 15 minutes. The samples were excited at 395 nm for NMM, 425 nm for ThT and 570 nm for ISCH-oa1. The emission spectra were acquired from 550 to 750 nm, 440 to 700 nm and 600 to 750 nm for NMM, ThT and ISCH-oa1, respectively. Data were collected every 2 nm at 25 °C with 5 nm entrance and exit slit widths. Raw ligand enhanced fluorescence spectra were first blanked with the corresponding sample spectra that resembled all chemical conditions except in the absence of the ligand. The blanked spectra were then smoothed over 10 nm (5 data points).^19^ All calculations mentioned were performed in Microsoft Excel.

### Reverse transcriptase stalling (RTS) assay

5 pmol of IVT RNA was added up to 4.5 μL with nuclease-free water, and 1 μL of 5 μM Cy5-labelled DNA primer was subsequently added. 3 μL reverse transcription buffer was added to give a final concentration of 150 mM LiCl/KCl, 4 mM MgCl_2_, 20 mM Tris pH 7.5, 1 mM DTT, and 0.5 mM dNTPs. The mixture was first heated to 75 °C for 3 min and then 35 °C for 5 min and 0.5 μL of Superscript III (200 U μL^–1^) (Thermo Fisher Scientific, USA) was added to make up the 10 μL reaction mixture. The reverse transcription was maintained at 50 °C for 15 min, and then 0.5 μL of 2 M NaOH was added at the end of the step. The temperature was immediately ramped up to 95 °C for 10 min. Finally, 10 μL of home-made 2× denaturing formamide dye was added to the reaction mixture.^36^ cDNAs were size fractionated using 8 M urea 8% denaturing polyacrylamide gel. The gel was scanned with Fujifilm FLA-9000.

### *In vitro* NAI modification

IVT RNA (5 pmol) was renatured at 95 °C for 1.5 min, then cooled to 4 °C for 1.5 min, and incubated at 37 °C in 150 mM KCl/LiCl, 0.5 mM MgCl_2_, and 40 mM Tris–HCl (pH 7.5). The final reaction volume was 19 μL. The reaction mixture was incubated at 37 °C, 10 min for system equilibration. NAI was then added to the reaction mixture at a final concentration of 0.05 M, and allowed to react with the RNA for 5 min at 37 °C. Dithiothreitol (DTT) was added to a final concentration of 0.2 M to quench the reaction, followed by RNA Clean & Concentrator-5 (Zymo Research, USA) to clean up the RNA.^18^ Minus NAI treatment was performed by adding anhydrous DMSO.

### In cell lysate NAI modification

One million cells were resuspended with 40 μL native cell lysis buffer (20 mM Tris–HCl pH 7.5, 100 mM KCl, 5 mM MgCl_2_, 0.5% Nonidet P-40) and 1 μL Riboblock RNase inhibitor (Thermo Fisher Scientific, USA). IVT RNA (200 pmol) was first denatured at 95 °C for 1.5 min, then cooled to 4 °C for 1.5 min for renaturation, and incubated at 37 °C in 200 mM KCl, and 10 mM Tris–HCl (pH 7.5). IVT RNA was added into the cell lysate and shaken at 37 °C for 5 min, and NAI (0.05 M final) was added and reacted for 5 min. The reaction was quenched by adding 4 μL of 2 M DTT and RNA was cleaned using a RNeasy Plus Kit (Qiagen, Germany) following the manufacturer's protocol. Minus NAI treatment was performed by adding anhydrous DMSO.

### Lithium ion-based primer extension (reverse transcription) for NAI probing

NAI-modified or untreated RNA was added up to 5.5 μL with nuclease-free water, and 1 μL of 5 μM Cy5-labelled DNA primer was then added. 3 μL of reverse transcription buffer was added to give a final concentration of 150 mM LiCl, 4 mM MgCl_2_, 20 mM Tris–HCl pH 7.5, 1 mM DTT, and 0.5 mM dNTPs. For dideoxy sequencing, 1 μL of 10 mM corresponding ddNTP was added to replace 1 μL of nuclease-free water. The mixture was heated at 75 °C for 3 min, followed by 35 °C for 5 min. Next, the 9.5 μL mixture was heated up to 50 °C, and 0.5 μL of Superscript III (200 U μL^–1^) (Thermo Fisher Scientific, USA) was added to make up the 10 μL reaction mixture. The reverse transcription was performed at 50 °C for 15 min, and then 0.5 μL of 2 M NaOH was added. The temperature was immediately raised to 95 °C for 10 min. Finally, 10 μL of home-made 2× denaturing formamide dye was added to the reaction mixture.^18^ The cDNAs were size fractionated using 8 M urea 8% denaturing polyacrylamide gel. The gel was scanned with Fujifilm FLA-9000.

### Synthesis of GTFH probes and characterization

The GFTH probe was synthesized according to the scheme in Chen and co-workers' paper.^34^ Mass spectra (MS) were recorded on a Shimadzu LCMS-2010A instrument with an ESI or ACPI mass selective detector, and high resolution mass spectra (HRMS) were recorded on a Shimadzu LCMS-IT-TOF. Flash column chromatography was performed with silica gel (200–300 mesh) purchased from Qingdao Haiyang Chemical Co. Ltd. The purity of the synthesized compound was confirmed to be higher than 95% by using analytical HPLC performed with a dual pump Shimadzu LC-20 AB system equipped with an Ultimate XB-C18 column (4.6 × 250 mm, 5 μm) and eluted with methanol–water (80 : 20) containing 0.1% TFA at a flow rate of 1.0 mL min^–1^. All chemicals were purchased from commercial sources unless otherwise specified. All the solvents were of analytical reagent grade and were used without further purification.

### Cell cultures and hybridization experiment in cells

The authenticated HeLa and HEK293T cells with no mycoplasma contamination were grown in DMEM media (Gibco, USA) containing 10% heat inactivated fetal bovine serum (Gibco, USA) at 37 °C, with 5% CO_2_ atmosphere. Cells were seeded in a glass bottom 96-well plate (MatTek, USA) and grew overnight. Oligonucleotide transfections were performed using 50 nM RNA oligonucleotides and Lipofectamine 3000 Transfection Reagent (Invitrogen, USA) for 4 h. Cells were fixed with 4% paraformaldehyde in DEPC–PBS at room temperature for 15 min. After rinsing with DEPC–PBS, cells were permeabilized in 0.5% TritonX-100/DEPC/PBS at 37 °C for 30 min. After rinsing with 2× SSC, probes were diluted at 0.3 μM in hybridization buffer (4× SSC, 0.5 mM EDTA, 10% dextran sulfate, 30% deionized-formamide in DEPC–H_2_O) and applied to the cells. Hybridization was done at 37 °C overnight. After hybridization, cells were washed in 2× SSC for 15 min twice and subsequently stained with (0.5 μg mL^–1^) DAPI for 15 min at 37 °C. For the RNase A treatment, cells were incubated with 200 units per mL^–1^ RNase A before hybridization at 37 °C for 1 h. Digital images were recorded using an LSM 710 laser scanning confocal microscope with a 63× objective lens, and analyzed with Imaris software (Bitplane Corp., UK). The Cellomics ArrayScan Vti (Thermo Fisher Scientific, USA) high-content imaging platform was used for the quantification of the fluorescence signal in cells. The high-content analysis automatically focused on the fluorescence channel of DAPI and captured the channel of ISCH-app.

### Live cell imaging

Cells were seeded in a glass bottom 96-well plate (MatTek, USA) and grew overnight. 10 μM RNA oligonucleotides were hybridized with a 10 μM ISCH-app rG4 imaging probe by heating at 95 °C for 5 min and cooling down to room temperature for 2 h. The mixture was then transfected into cells using Lipofectamine 3000 Transfection Reagent (Invitrogen, USA) for over 4 h. Digital images were recorded using an Olympus FV3000 laser scanning confocal microscope with a 63× objective lens, and analyzed with Imaris software (Bitplane Corp., UK).

### Transfection and luciferase reporter assay

The wildtype and mutant APP 3′UTR DNA sequences were ligated to the firefly/Renilla luciferase reporter vector, psiCHECK-2 (Promega, USA), between NotI and XhoI restriction enzyme sites at the 3′ end of the Renilla luciferase gene by IGENE (China). Authenticated HEK293T/HeLa cells with no mycoplasma contamination were seeded in 96-well black-wall optical plates (PerkinElmer, USA) at the density of 50 000 cells per well. In each well, the cells were transfected with 10 ng luciferase reporter plasmid using Lipofectamine® 2000 according to the manufacturer's protocol (Thermo Fisher Scientific, USA). After 5 and 24 hours, the medium was replaced with fresh medium. The cells were harvested 48 hours after transfection using the Dual Luciferase Reporter Assay System (Promega, USA) according to the manufacturer's manual. Firefly and Renilla luciferase activities were determined using the Synergy H1 microplate reader (Biotek, USA). Renilla luciferase activity was normalized to firefly luciferase activity (firefly luciferase is constitutively expressed by psiCHECK-2 vector as an internal control) and presented as the average of three transfection repeats.

### RNA extraction and qRT-PCR

Total RNA was extracted, cleaned and concentrated from cells using RNase Plus Mini Kit (Qiagen, Germany) according to the manufacturer's manuals. RNA samples were quantified using NanoDrop analysis (Thermo Fisher Scientific, USA). 100 ng total RNA was converted to cDNA with the Superscript III first-strand synthesis system (200 U μL^–1^) (Thermo Fisher Scientific, USA) by reverse transcription. qRT-PCR analysis of mRNAs was performed using an SsofastTM Evagreen (SYBR Green) qPCR master mix (Bio-Rad). All qPCR reactions were performed by using a CFX96 Touch™ Real-Time PCR Detection System (Bio-Rad, USA).

### Statistical analysis

In all graphs, data are presented as mean ± standard error of the mean (SEM). For quantification, each experiment was usually repeated at least three times with cells from three cell passages.

## Conflicts of interest

The authors declare no competing financial interests.

## Supplementary Material

Supplementary information
