# Supplementary material for: Structural analysis and cellular visualization of APP RNA G-quadruplex
Source: Chem Sci. 2019 Oct 29;10(48):11095–102. doi: 10.1039/c9sc02768h (PMC7069244; doi:10.1039/c9sc02768h)
Supplement: Supplementary file 1 [file SC-010-C9SC02768H-s001.pdf]

Electronic Supplementary Material (ESI) for Chemical Science.
This journal is © The Royal Society of Chemistry 2019

Supplementary Information

Table S1. Oligonucleotides used in this study.

Figure S1. CD-detected titration of *APP* 3'UTR rG4wt and rG4mut motif.

Figure S2. UV-detected melting of *APP* 3'UTR rG4wt and rG4mut motif.

Figure S3. Concentration-dependent UV melting of *APP* 3'UTR rG4wt motif.

Figure S4. Intrinsic fluorescence titration of *APP* 3'UTR rG4wt and rG4mut motif.

Figure S5. Fluorescence turn-on assays on *APP* 3'UTR rG4mut region with G-quadruplex-specific ligands.

Figure S6. RTS and SHALiPE assays on *APP* 3'UTR rG4mut region.

Figure S7. SHALiPE assay on *APP* 3'UTR rG4wt region in cell lysate.

Figure S8. Design of G-rich RNA sequences and the ISCH-app and FAM-app probes used in this study.

Figure S9. ISCH-app GTFH probe synthesis.

Figure S10. ESI mass spectrum of ISCH-app.

Figure S11. Cell imaging and statistical analysis on *APP* 3'UTR rG4wt and rG4mut region in HeLa cells.

Figure S12. Live cell imaging and statistical analysis on *APP* 3'UTR rG4wt and rG4mut region in HeLa cells.

Figure S13. Reporter gene assay and qPCR result on *APP* rG4wt, G2 and rG4mut plasmid on HeLa cells..

Figure S14. CD-detected titration, UV-detected melting and intrinsic fluorescence titration of *APP* G2 rG4 motif region.

Table S1. Oligonucleotides used in this study.

**Name**	**Sequence (5'-3')**
APP rG4wt motif	**GGGGCGGGUGGGGAGGGG**
APP rG4mut motif	GAAGCGAGUGAAGAGAAG
APP G2 motif	AAGGCAGGUAGGAAGGAA
APP rG4wt region	GGUCUUCGGACCAAUUGGGUCUUUGAUAAAGAAAAGAAUCCCUGUUCAUUGUAAGCACUUUUAC**GGGGCGGGUGGGGAGGGG**UGCUCUGCUGGUCUUCAAUUACCAAGAAUUCUCCAAAACAAUUUUCUGCAGGAUGAUUGUACAAAUCGCGCUUCGGUGCGGUUC
APP rG4mut region	GGUCUUCGGACCAAUUGGGUCUUUGAUAAAGAAAAGAAUCCCUGUUCAUUGUAAGCACUUUUACGAAGCGAGUGAAGAGAAGUGCUCUGCUGGUCUUCAAUUACCAAGAAUUCUCCAAAACAAUUUUCUGCAGGAUGAUUGUACAAAUCGCGCUUCGGUGCGGUUC
Cy5-labelled reverse transcription primer	GAACCGCACCGAAGCGCG
APP rG4wt DNA inserted in vector	CTCGAGTTGGGTCTTTGATAAAGAAAAGAATCCCTGTTCATTGTAAGCACTTTTAC**GGGG**C**GGG**T**GGGG**A**GGGG**TGCTCTGCTGGTCTTCAATTACCAAGAATTCTCCAAAACAATTTTCTGCAGGATGATTGTAGCGGCCGC
APP rG4mut DNA inserted in vector	CTCGAGTTGGGTCTTTGATAAAGAAAAGAATCCCTGTTCATTGTAAGCACTTTTACGAAGCGAGTGAAGAGAAGTGCTCTGCTGGTCTTCAATTACCAAGAATTCTCCAAAACAATTTTCTGCAGGATGATTGTAGCGGCCGC
APP G2 DNA inserted in vector	CTCGAGTTGGGTCTTTGATAAAGAAAAGAATCCCTGTTCATTGTAAGCACTTTTACAAGGCAGGTAGGAAGGAATGCTCTGCTGGTCTTCAATTACCAAGAATTCTCCAAAACAATTTTCTGCAGGATGATTGTAGCGGCCGC
Renilla luciferase forward primer	ACAAGTACCTCACCGCTTGG
Renilla luciferase reverse primer	GACACTCTCAGCATGGACGA
Firefly luciferase forward primer	GGACATCACCTATGCCGAGT
Firefly luciferase reverse primer	GTTCTCAGAGCACACCACGA
ISCH-app rG4 imaging probe	ISCH-oa1-GTAAAAGTGCTTACAATGAACAGGG
FAM-app RNA imaging probe	FAM-TTGGTAATTGAAGACCAGCAGAGCA

Footnote: The Gs involved in rG4 in *APP* 3'UTR RNA/DNA are shown in bold. The rG4 mutant is designed by substituting some Gs with As (underlined). For structure probing, the Cy5-labelled primer and its complementary sequence in RNA is shown in blue. The 5' linker is shown in dark red. For cell imaging, the ISCH probe and its complementary sequence in RNA is in purple while the FAM labelled probe and corresponding RNA sequence is in green. The XhoI restriction site is in yellow and the NotI restriction site is in gray. The *APP* G2 is designed by substituting some Gs with As (underlined).

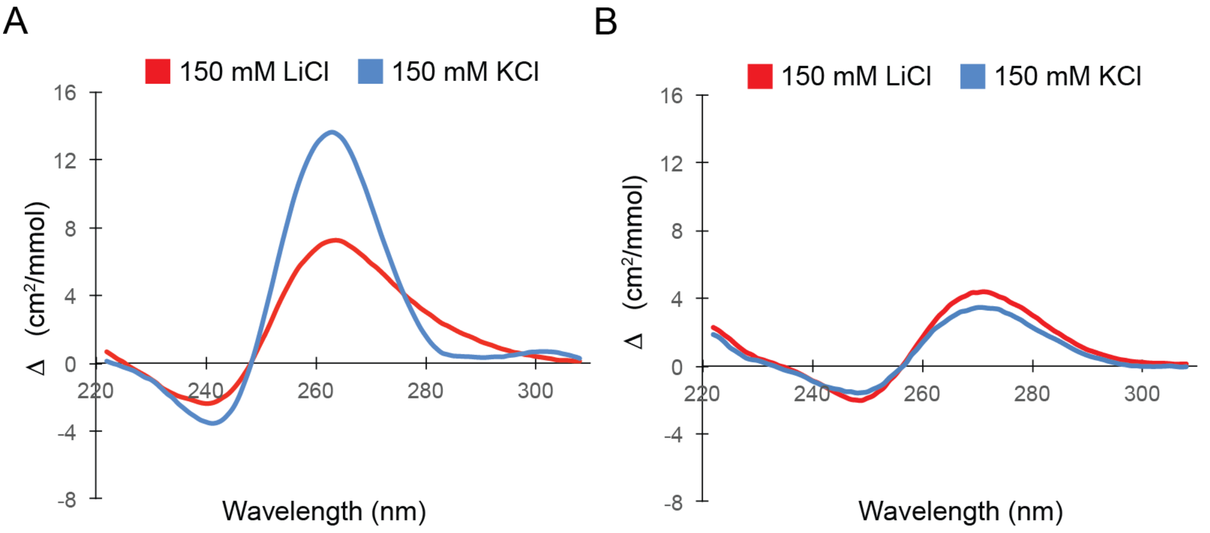

Figure S1. CD-detected titration of *APP* 3'UTR rG4wt and rG4mut motif. A) CD-detected titration scanned from 220-310 nm at 25 °C under KCl (Blue) and LiCl (Red) conditions for *APP* 3'UTR rG4wt motif. The positive and negative peak at ~262 nm and 240 nm respectively, as well as the K^+-dependent CD signal are signature of parallel topology rG4, suggesting that the G-rich region of *APP* 3'UTR rG4wt motif forms a parallel topology rG4. B) CD-detected titration scanned from 220-310 nm at 25 ˚C under KCl (Blue) and LiCl (Red) conditions for *APP* 3'UTR rG4mut motif. No obvious changes were observed among KCl and LiCl conditions.

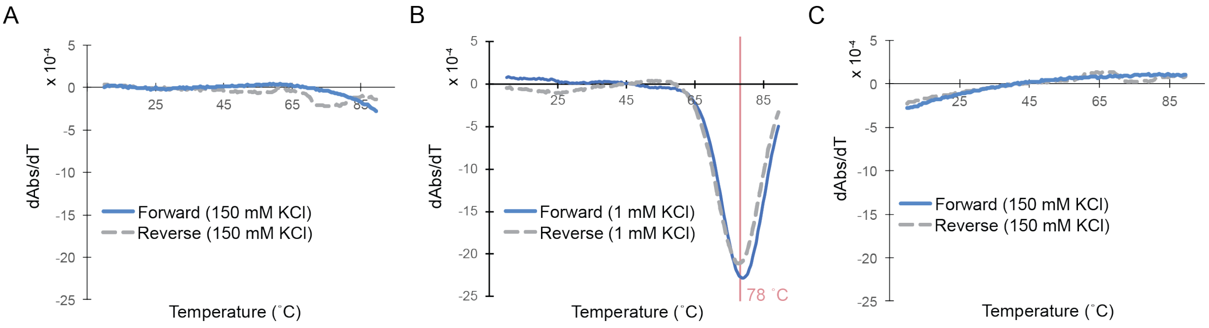

Figure S2. UV-detected melting of *APP* 3'UTR rG4wt and rG4mut motif. A) UV-detected melting monitored at 295 nm under 150 mM KCl. The melting temperature of the *APP* 3'UTR rG4wt motif is ≥ 95 °C. Solid blue line indicates forward scan, and dotted grey line indicates the reverse scan. B) UV-detected melting monitored at 295 nm under 1 mM KCl and the melting temperature of the *APP* 3'UTR rG4wt motif was determined to be 78 °C. Solid blue line indicates forward scan, and dotted grey line indicates the reverse scan. C) UV-detected melting monitored at 295 nm and no distinct rG4 melting profile was observed for *APP* 3'UTR rG4mut motif. Solid blue line indicates forward scan, and dotted grey line indicates the reverse scan.

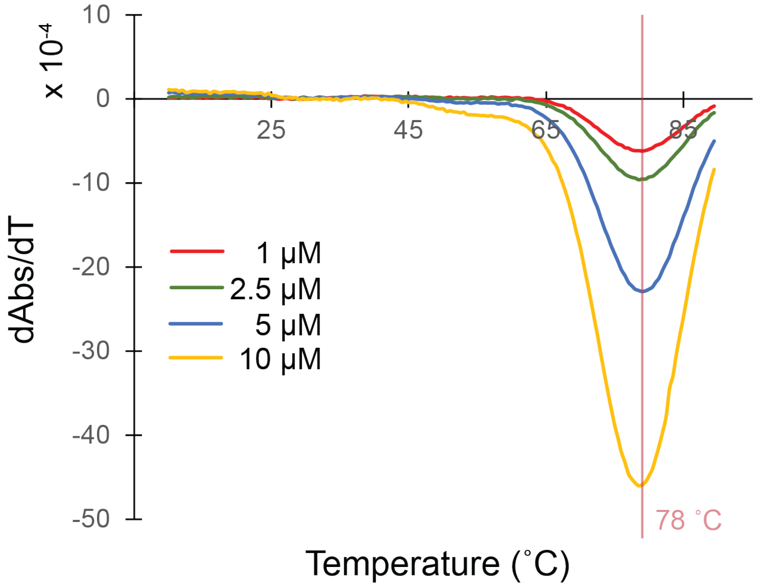

Figure S3. Concentration-dependent UV melting of *APP* 3'UTR rG4wt motif. The melting temperatures obtained from the 1 mM KCl UV-melting profiles of the *APP* 3'UTR rG4wt motif in 1 (red), 2.5 (green), 5 (blue), and 10 µM (yellow) concentrations were 78 °C regardless of oligo concentration. It was observed that the melting temperature of the oligo was independent of the oligo concentration, suggesting the *APP* 3'UTR rG4wt motif was folded intramolecularly.

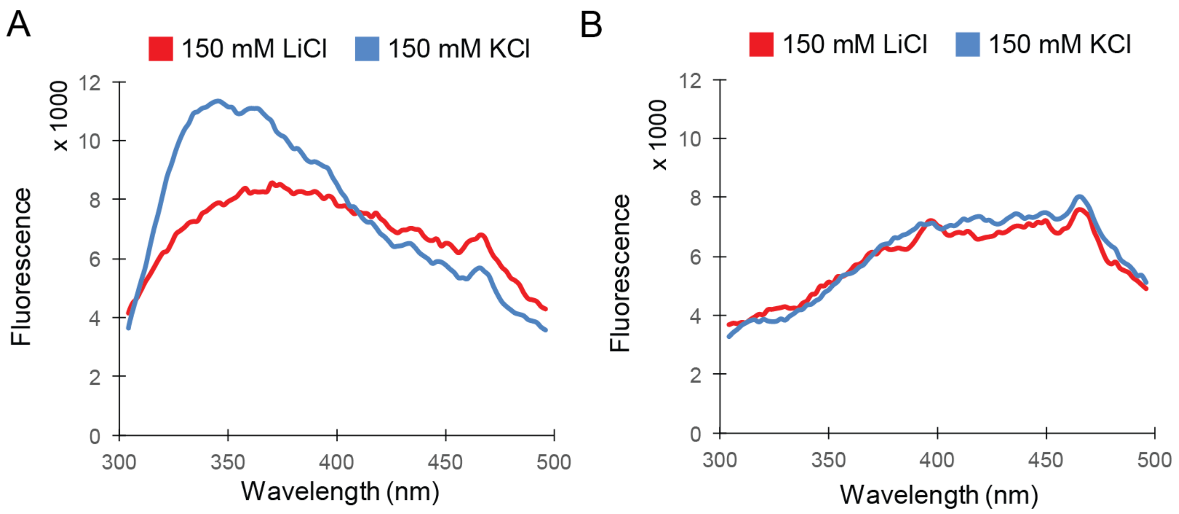

Figure S4. Intrinsic fluorescence titration of *APP* 3'UTR rG4wt and rG4mut motif. A) Intrinsic fluorescence titration in 10 mM LiCac (pH 7.0) and 150 mM KCl (Blue) and LiCl (Red) at 25 °C. From this data, the signal changes between KCl and LiCl condition, suggesting rG4 formation in *APP* 3'UTR rG4wt motif. The λ_{em} was determined to be 346 nm under KCl condition. B) Intrinsic fluorescence titration in 10 mM LiCac (pH 7.0) and 150 mM KCl (Blue) and LiCl (Red) at 25 °C. From this data, no difference in intrinsic fluorescence was detected for *APP* 3'UTR rG4mut motif.

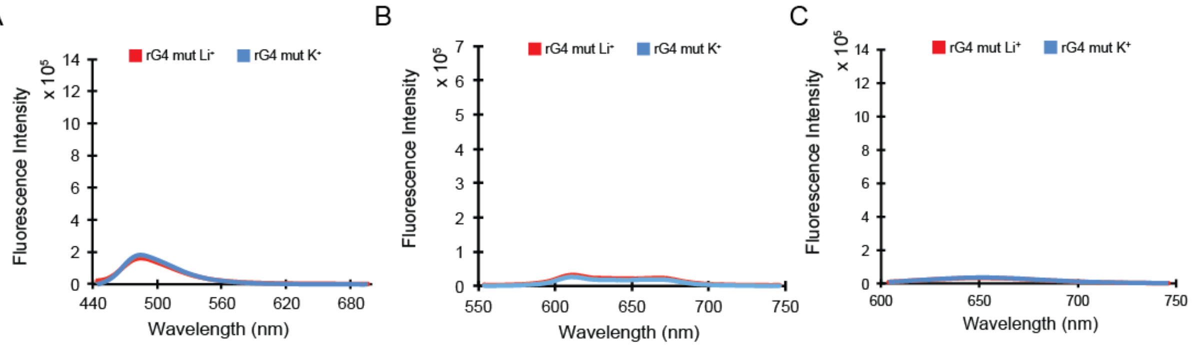

Figure S5. Fluorescence turn-on assays on *APP* 3'UTR rG4mut region with G-quadruplex-specific ligands. A) ThT ligand-enhanced fluorescence on *APP* 3'UTR rG4mut region in 150 mM LiCl and KCl, respectively. B) NMM ligand-enhanced fluorescence on *APP* 3'UTR rG4wt region in 150 mM LiCl and KCl, respectively. C) ISCH-oa1 ligand-enhanced fluorescence on *APP* 3'UTR rG4wt region in 150 mM LiCl and KCl, respectively. For all three cases, the spectra under Li^+ and K^+ are highly similar, suggesting no rG4 formation under such condition.

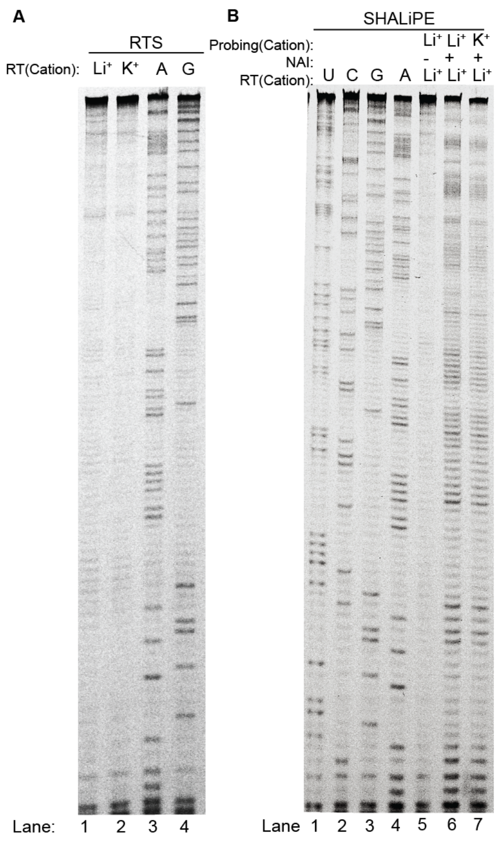

Figure S6. RTS and SHALiPE assays on *APP* 3'UTR rG4mut region. A) RTS assay on *APP* 3'UTR rG4mut region. In lane 1 and 2, 150 mM Li^+ and 150 mM K^+, respectively, was used for reverse transcription. Lane 3 and 4 are ladder by dideoxynucleoside sequencing. No stalling was observed in this RNA. B) SHALiPE assay on *APP* 3'UTR rG4mut region. *APP* 3'UTR rG4mut region was probed with DMSO (control) or with NAI under Li^+ and K^+ (lane 5-7). Lanes 1-4 are dideoxy sequencing. Under physiological K^+ conditions (lane 7), *APP* 3'UTR rG4mut region displayed similar NAI profiles comparing with Li^+ conditions (lane 6), suggesting no rG4 is present in *APP* 3'UTR rG4mut region.

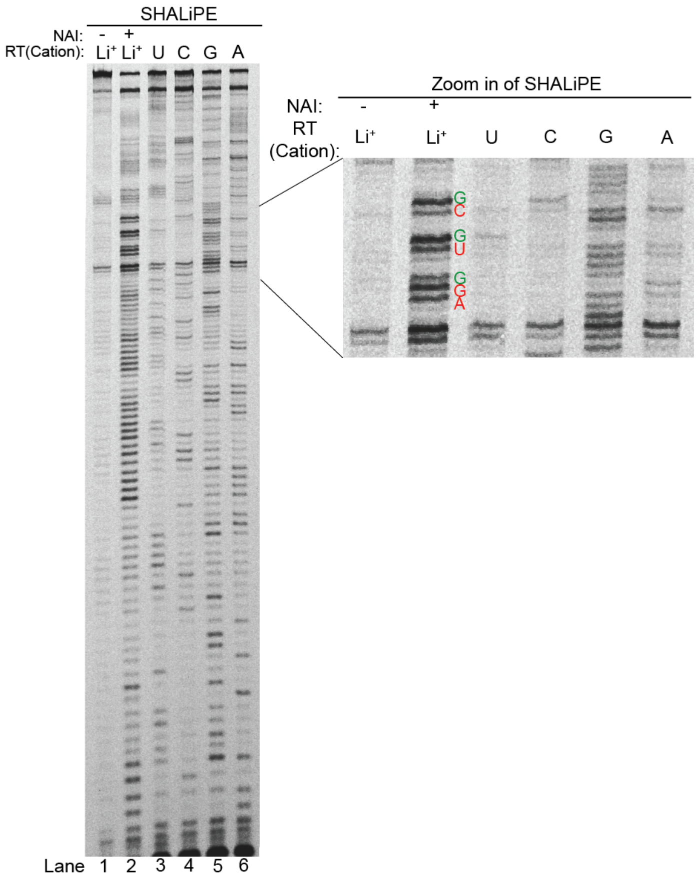

Figure S7. SHALiPE assay on *APP* 3’UTR rG4wt region in cell lysate. *APP* 3’UTR rG4wt region was probed with DMSO or NAI (see methods). The rG4 at the *APP* 3’UTR rG4wt region displayed NAI profiles (lanes 2) that are similar to K^+ condition *in vitro* than Li^+ condition *in vitro* (compare with Figure 3B). On the right is the zoom in of rG4 motif from the SHALiPE gel. From the band intensity, the nucleotides of the loops of rG4 (red), and the G nucleotides at the 3’ of each G-tracts were highly modified (green).

APP 3’UTR mRNA 166NT

G-rich Sequence *Tail Sequence(TS)*

5’-UUGGGUCUUUGAUAAAGAAAAGAAUCCCUGUUCAUUGUAAGCACUUUUAC
GGGGCGGGUGGGGAGGGGUGCUCUGCUGGUCUUCAAUUACCAAGAAUUCUC
CAAAACAAUUUUCUGCAGGAUGAUUGUACAAAUCGCGCUUCGGUGCGGUUC-3’

Probes

Fluorescent Label for Detection *Anti-Tail Sequence(ATS)*

G-quadruplex Light up ISCH-oa1-3’-GGGACAAGTAACATTCGTGAAAATG-5’

Marker FAM-3’-ACGAGACGACCAGAAGTTAATGGTT-5’

Figure S8. Design of G-rich RNA sequences and the ISCH-app and FAM-app probes used in this study. Sequence in pink is G-rich sequence, which was predicted to form G-quadruplex *in vivo*. Sequence in purple and in green are attached to ISCH-oa1 and FAM label on the 3’ end, respectively.

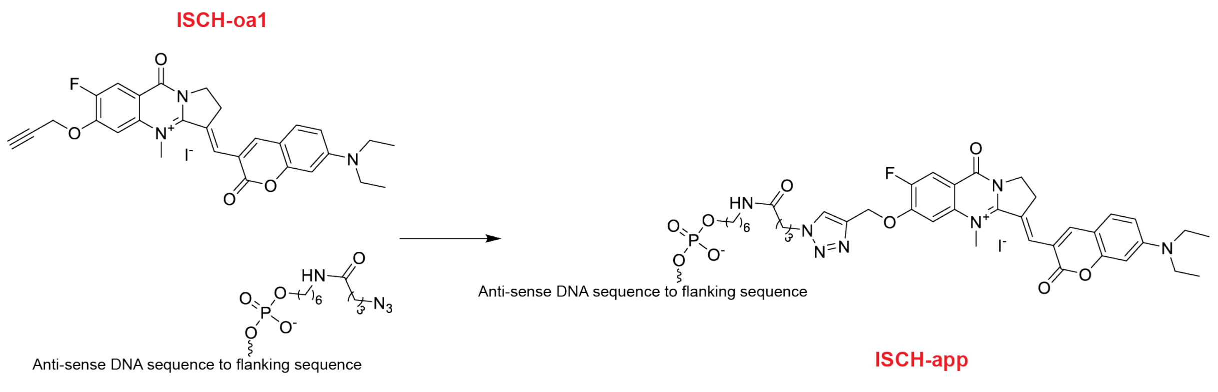

Figure S9. ISCH-app GTFH probe synthesis. ISCH-oa1 (1 mM) and azido-modified DNA oligonucleotide (0.05 mM) were mixed in water (200 µL) containing fresh sodium ascorbate (1.2 mM), copper sulfate (0.6 mM) and was stirred at reflux for 24 h at 37°C. The purity was determined by RP-HPLC-UV and mass spectrometry.

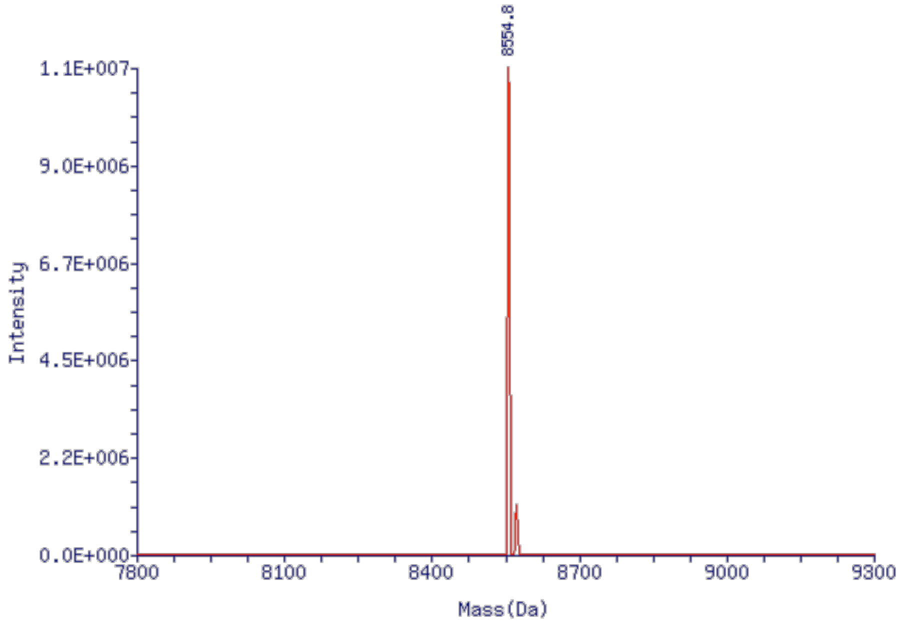

Figure S10. ESI mass spectrum of ISCH-app. Targeted size is at 8554.0Da and the observed size is at 8554.8Da.

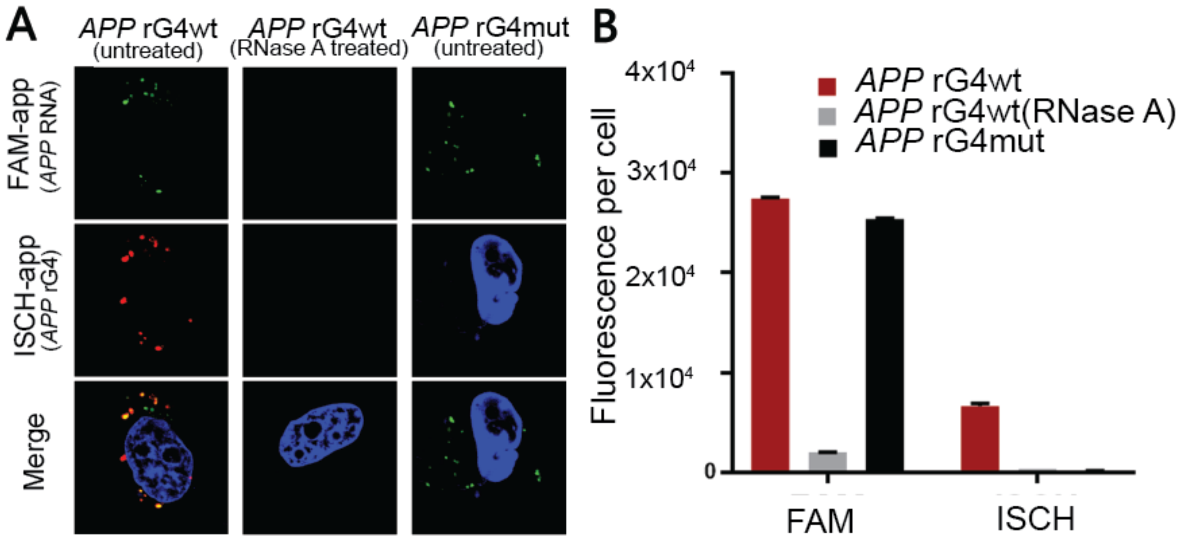

Figure S11. Cell imaging and statistical analysis on *APP* 3'UTR rG4wt and rG4mut region in HeLa cells. A) Cell imaging shows the imaging result under confocal microscope for both FAM labelled probe (FAM-app) and GFTH probe (ISCH-app). RNase A was added for control. B) Quantification of ISCH-app spots and FAM-app spots in cells. The data were acquired from 10,000 cells per sample and three independent experiments were performed.

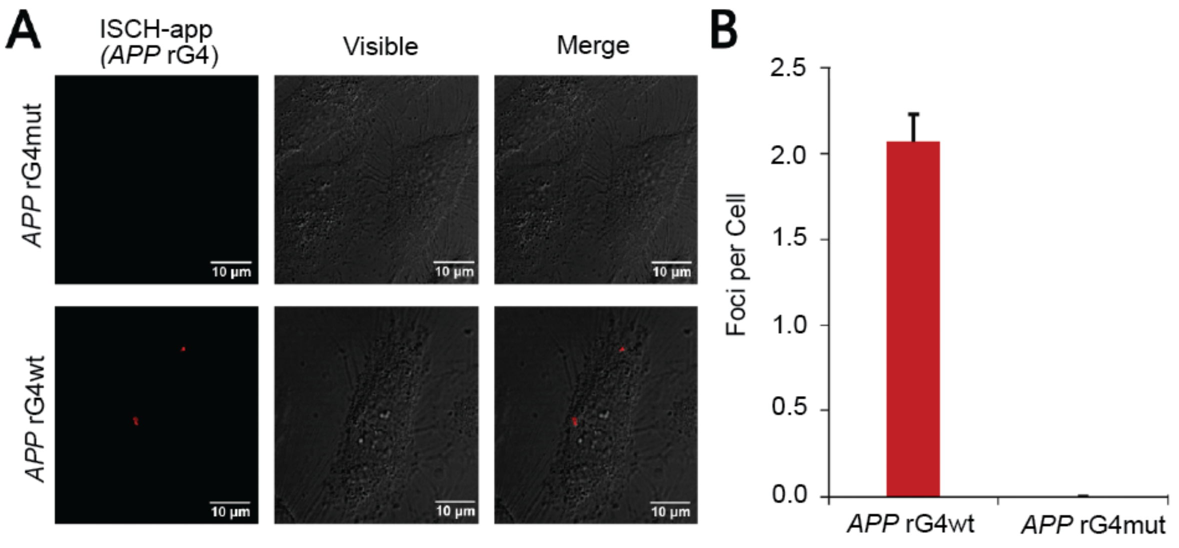

Figure S12. Live cell imaging and statistical analysis on *APP* 3'UTR rG4wt and rG4mut region in HeLa cells. A) Digital images of live cell under confocal microscope on *APP* 3'UTR rG4wt and rG4mut region with GFTH probe (ISCH-app). B) Quantification for ISCH-app foci per cell. The data were acquired from 100 cells per sample.

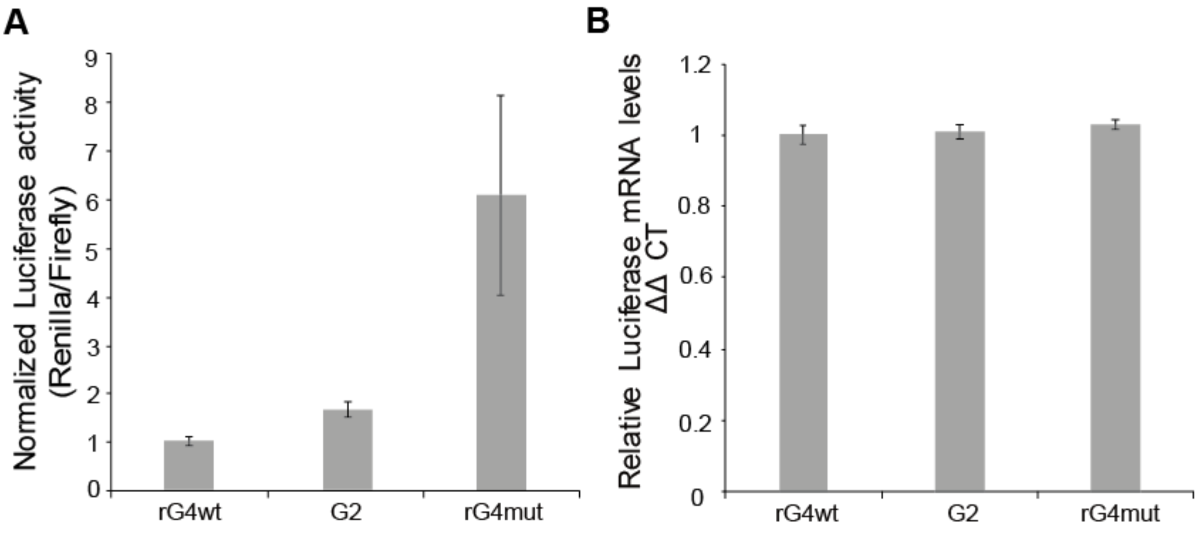

Figure S13. Reporter gene assay and qPCR result on *APP* rG4wt, G2 and rG4mut plasmid on HeLa cells. A) Average normalized luciferase activity was acquired 2 days after transfection. B) qRT-PCR result on *APP* rG4wt, G2 and rG4mut plasmid. Values were obtained from 3 biological replicates and errors bars display standard error of mean.

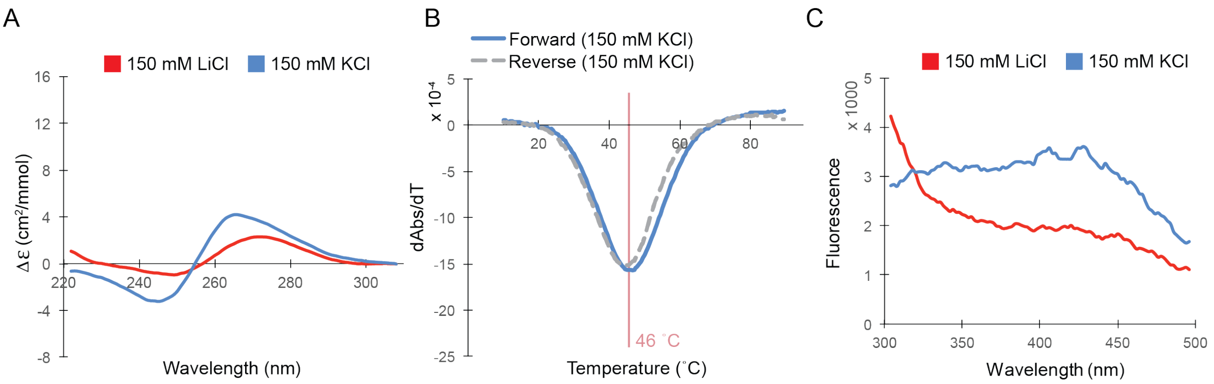

Figure S14. CD-detected titration, UV-detected melting and intrinsic fluorescence titration of *APP* G2 rG4 motif region. A) CD-detected titration scanned from 220-310 nm at 25 °C under KCl (Blue) and LiCl (Red) conditions. The positive and negative peak at ~262 nm and ~240 nm, as well as the K^+-dependent CD signal are signature of parallel topology rG4, suggesting that the G-rich region of *APP* G2 rG4 motif forms a parallel topology rG4. B) UV-detected melting monitored at 295 nm and the melting temperature was determined to be 46 °C under 150 mM KCl. Solid blue line indicates forward scan, and dotted grey line indicates the reverse scan. C) Intrinsic fluorescence titration in 10 mM LiCac (pH 7.0) and 150 mM KCl (Blue) and LiCl (Red) at 25 °C. From this data, the rG4 forms and the λ_{em} was determined to be 428 nm under KCl condition.
